# Case report: Cardiac neuroendocrine carcinoma and squamous cell carcinoma treated with MR-guided adaptive stereotactic radiation therapy

**DOI:** 10.3389/fonc.2024.1411474

**Published:** 2024-09-16

**Authors:** Xinru Chen, Julius K. Weng, Angela Sobremonte, Belinda M. Lee, Neil W. Hughes, Mustefa Mohammedsaid, Yao Zhao, Xiaochun Wang, Xiaodong Zhang, Joshua S. Niedzielski, Sanjay S. Shete, Laurence E. Court, Zhongxing Liao, Percy P. Lee, Jinzhong Yang

**Affiliations:** ^1^ Department of Radiation Physics, The University of Texas MD Anderson Cancer Center, Houston, TX, United States; ^2^ The University of Texas MD Anderson Cancer Center UTHealth Houston Graduate School of Biomedical Sciences, Houston, TX, United States; ^3^ Department of Radiation Oncology, The University of Texas MD Anderson Cancer Center, Houston, TX, United States; ^4^ Department of Radiation Therapeutic Physics, The University of Texas MD Anderson Cancer Center, Houston, TX, United States; ^5^ Department of Biostatistics, The University of Texas MD Anderson Cancer Center, Houston, TX, United States; ^6^ Department of Radiation Oncology, City of Hope Orange County, Lennar Foundation Cancer Center, Irvine, CA, United States

**Keywords:** cardiac tumor, cardiac metastasis, MR-guided radiotherapy, SBRT, MR-Linac

## Abstract

We present two cases of cardiac metastases adjacent to the right ventricle in a 55-year-old male and a 61-year-old female, both treated with magnetic resonance (MR)-guided adaptive stereotactic radiation therapy (SBRT). The prescribed regimen was 30Gy delivered in 3 fractions using a 1.5 Tesla magnetic resonance linear accelerator (MR-linac). Patients exhibited favorable tolerance to the treatment, with no observed acute toxicity.

## 1 Introduction

Cardiac tumors, though rare in incidence, present substantial challenges in both diagnostic and therapeutic treatment options. The incidence of primary cardiac tumors is reported to range from 0.002% to 0.3%, whereas secondary cardiac tumors, or cardiac metastases, exhibit a markedly higher frequency, being 22 to 132 times more common (1, 2). Although biopsy remains the gold standard for diagnosing cardiac tumors, the integration of multimodality imaging, encompassing transthoracic echocardiography, cardiovascular magnetic resonance (MR), cardiac computed tomography (CT), and positron emission tomography (PET/CT), enables accurate diagnosis and treatment strategy formation, often obviating the need of invasive biopsy (3). Notably, primary lung cancer, breast cancer, and hematologic malignancies have elevated propensities for cardiac involvement (4). In contrast to the imperative for complete resection in many primary cardiac tumors, the management of cardiac metastasis is highly individualized and contingent upon the nature of the primary tumor (3). Surgical debulking is reserved for instances involving intracavitary metastases causing cardiac decompensation or solitary disease with a favorable prognosis. Nevertheless, the overall survival rate for patients with malignant cardiac tumors remains bleak. Historically, radiation therapy has served as a palliative treatment to alleviate symptoms and improve the quality of life for patients (5). In recent years, stereotactic body radiation therapy (SBRT) has emerged as an alternative definitive treatment method, particularly for patients that are inoperable, delivering higher and more definitive dose (6, 7). Moreover, the advent of MR-guided radiotherapy has significantly advanced treatment precision through superior soft-tissue/tumor visualization and the ability for daily plan adaptation. This report presents two cases of cardiac metastases treated at our institution on a high-field 1.5T MR-Linac using adaptive SBRT.

## 2 Methods and materials

**Figure 1:**
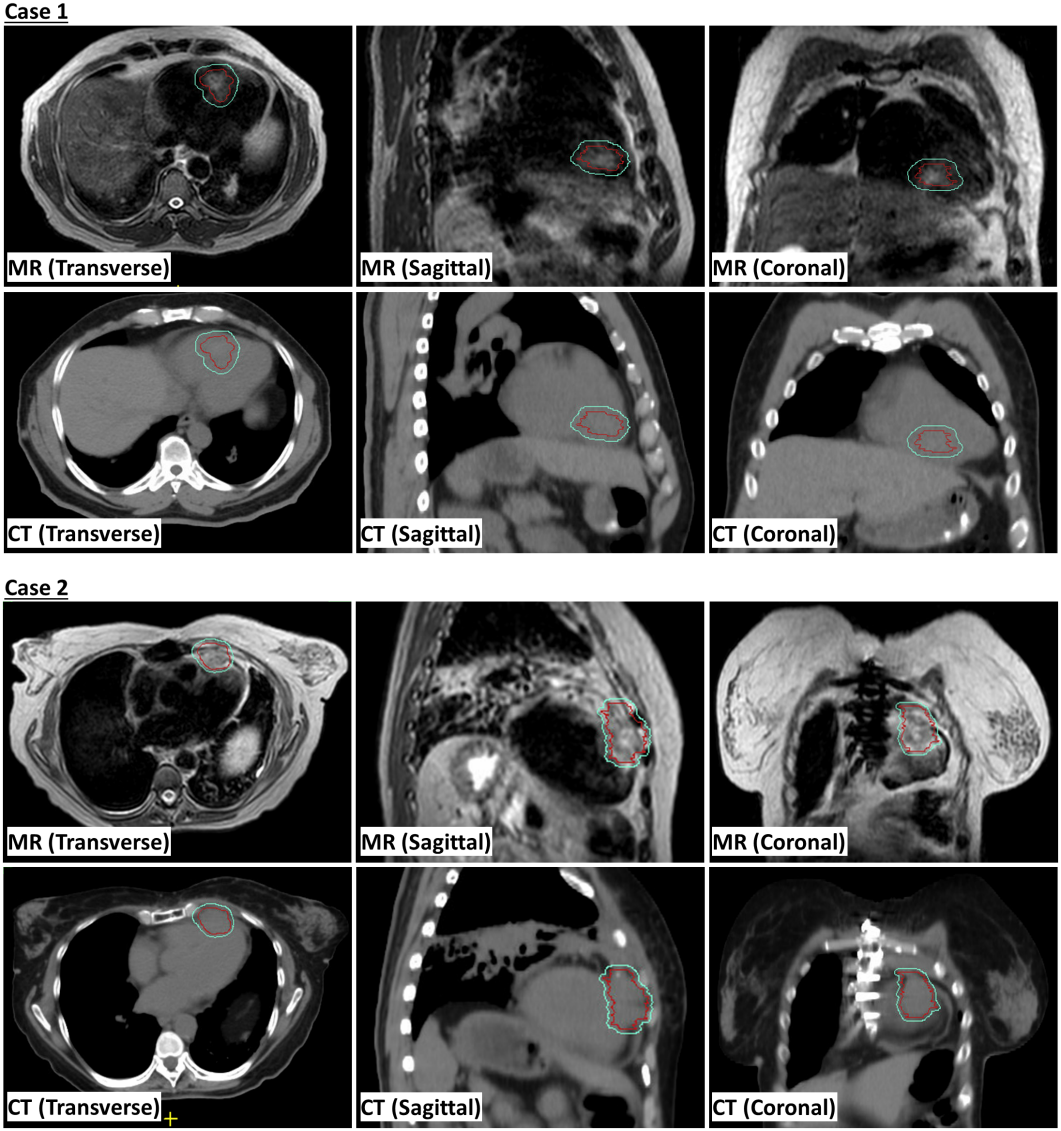
Magnetic resonance (MR) and computed tomography (CT) scans showing internal gross tumor volume (iGTV) in red and planning risk volume (PTV) in cyan.

This study was approved by the local Institutional Review Board (protocol # 2022-0521). Eligible patients with cardiac metastases were enrolled in the treatment protocol following a comprehensive discussion by a multi-disciplinary team, which considered the treatment history, the benefits and risks of potential interventions. A 4DCT simulation scan was conducted in the supine position, arms raised up, using a Wing Board and Vac-lock bag. This scan served to estimate the respiratory and cardiac motion, acquire electron density data, and delineate organs-at-risk (OARs). MR simulation was executed the same day of 4DCT simulation on a 1.5 Tesla Unity MR-linac system, using T2-weighted transverse scanning under free-breathing conditions. The internal gross target volume (iGTV) was contoured based on the T2-weighted scans to account for cardiac motion. To verify the contour integrity, contrast CT images were fused with the T2-weighted MR image. Semi-automatic tools were used to contour OARs on 4DCT scans, and both OAR contours and electron densities were subsequently transferred to the MR image. The synthetic CT generated from the MR image underwent subjective verification. The planning target volume (PTV) was generated through isotropic expansion of the iGTV with a margin based on the estimated respiratory motion. Demonstration of iGTV and PTV on MR and CT scans is shown in **Figure 1**. A reference plan, prescribing 30 Gy to 95% of the PTV, was created on the simulation T2-weighted image. Patients received treatment on consecutive days with free-breathing conditions. Daily adaptation of the reference plan employed the adapt-to-position (ATP) technique, where an isocenter shift is made on the reference plan based on rigid registration followed by plan re-optimization (8), given minor anatomical changes observed during the whole treatment course.

## 3 Case presentation

**Figure 2:**
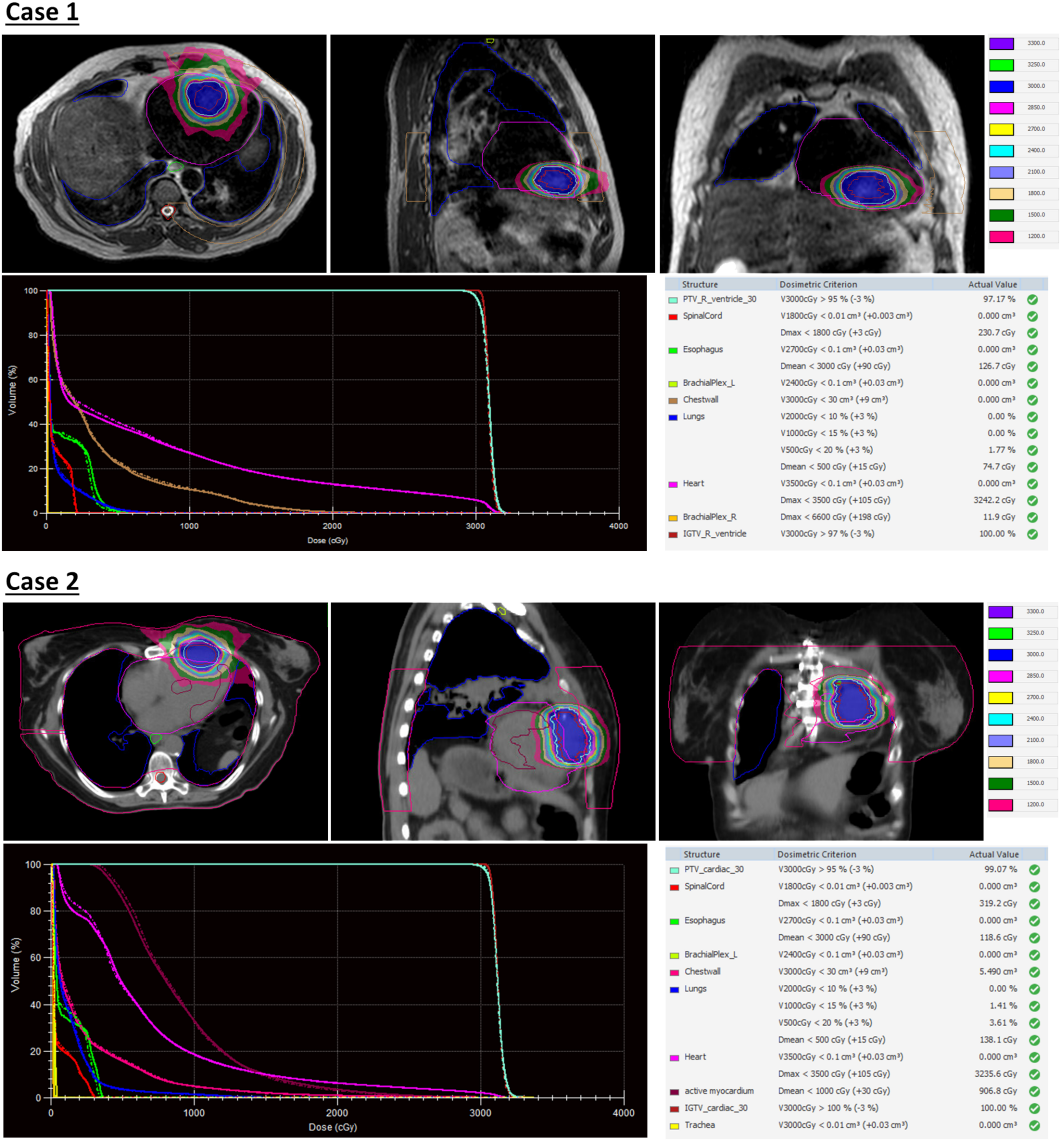
Dose distribution and dose-volume histograms (DVH) of the third fraction of stereotactic body radiation therapy (SBRT) treatment. In the DVH, solid curves represent the adapted plan and dash curves represent the reference plan created on simulation MR images.

**Figure 3:**
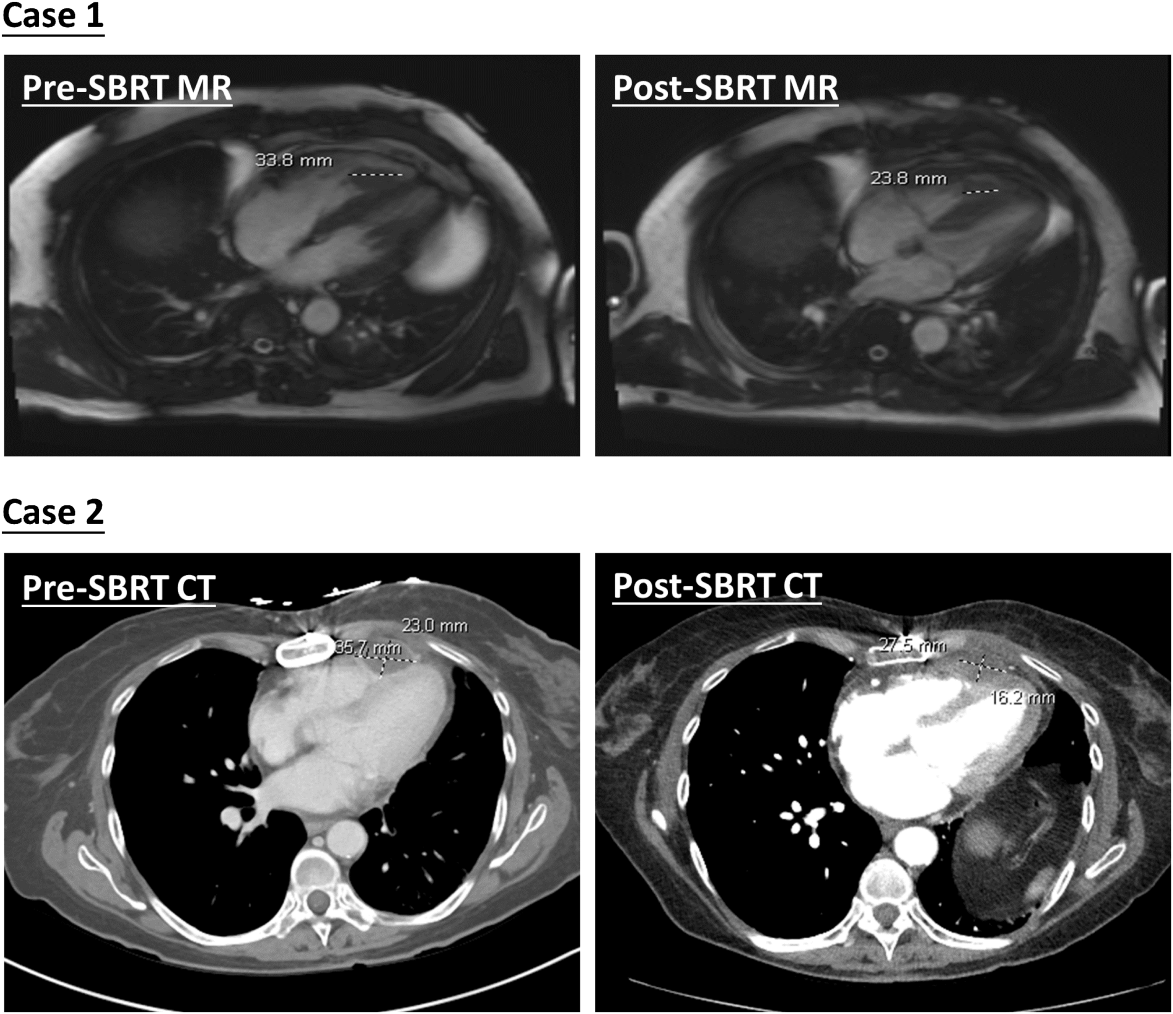
Demonstration of pre- and post-SBRT cardiac lesions. Diagnostic MR and CT images were both acquired with contrast agents.

The first case is a 55-year-old male initially diagnosed with bulky stage II T2N0M0 laryngeal cancer in May 2018. The patient underwent chemoradiation treatment, receiving a total dose of 70Gy (2Gy per fraction) with concomitant cis-platinum. In 2020, he presented with left arm subcutaneous nodules and metastatic recurrence in the larynx. Subsequent biopsy of the subcutaneous nodules indicated poorly differentiated neuroendocrine carcinoma. Palliative radiation was then administered to multiple subdermal sites in 2020 in conjunction with chemo- and targeted therapy (carboplatin/etoposide/atezolizumab) and immunotherapy (Durvalumab) in 2021. By October 2021, the patient progressed with an intracardiac mass. A chest CT with contrast revealed an enlarging nodule in the right ventricle. Multi-sequence multiplanar cardiac MR imaging, acquired with and without intravenous contrast, identified a mass in the right ventricle lumen measuring 34 mm in greatest diameter. The patient claimed generalized pains in previously treated sites but no cardiovascular symptoms. Following consultation with the multi-disciplinary team, the patient was referred to the Department of Radiation Oncology for treatment with SBRT on MR-linac in November 2021. The iGTV is 12.45 cm^3^ with the consideration of cardiac and respiratory motion. The PTV was created by expanding the iGTV by 5mm in all directions. The total dose of 30 Gy was prescribed in 3 fractions, targeting at least 95% of the PTV volume. Detailed patient and treatment characteristics are shown in **Table 1**. The dosimetric criteria are demonstrated in **Table 2**. Throughout the treatment course, the patient continued to take Xarelto 10mg orally once daily to prevent blood clots. The daily plan was re-optimized using ATP technique. The dose distribution and dose-volume histograms (DVH) of the third fraction are shown in **Figure 2**. After the SBRT treatment of the cardiac mass, the patient received chemotherapy (carboplatin/docetaxel) for subcutaneous metastases in January 2022. Three months post the SBRT treatment, the patient reported subdermal pains and moderate fatigue, with no chest pain and tightness. Four months post the treatment, a PET/CT scan suggested a reasonable response to SBRT for his cardiac metastasis. Follow-up cardiac MR with and without contrast, along with chest CT with contrast after 5 months, indicated a reduction in size of the cardiac mass, as shown in **Figure 3**. Another PET/CT scan acquired 7 months later suggested a stable response at the treatment site. However, he had further progression of existing pulmonary, intramuscular, and subcutaneous metastases.

**Table 1:** Patient and treatment characteristics.

	Case 1	Case 2
Age	55	61
Gender	Male	Female
Histology	Neuroendocrine carcinoma	Squamous cell carcinoma
Site of tumor	Right ventricle septa wall	Anterior right ventricle
Prescription dose (Gy)	30	30
Number of fractions	3	3
Isodose prescription (PTV)	95%	95%
iGTV volume (cc)	12.45	21.59
Minimum iGTV dose	29.89	30.12
Max iGTV dose	32.14	32.99
PTV margin (mm)	5	3
PTV volume (cc)	38.53	40.07
Minimum PTV dose	28.64	29.21
Max PTV dose	32.38	32.99
PTV coverage	96.99%	99.57%
Monitor units	2267.4	2632.15
Beams	11	11
Segments	62	82

**Table 2:** Dosimetric criteria of targets and organs-at-risk (OARs) for the two cases.

Structure	Case 1		Case 2	
	Constraint	Planned	Constraint	Planned
iGTV	V3000cGy > 97%	99.98%	V3000cGy > 100%	100.00%
PTV	V3000cGy > 95%	96.99%	V3000cGy > 95%	99.57%
Spinal Cord	V1800cGy < 0.01 cm^3^	0.0 cm^3^	V1800cGy < 0.01 cm^3^	0.0 cm^3^
	Dmax < 1800 cGy	225.0 cGy	Dmax < 1800 cGy	301.8 cGy
Esophagus	V2700cGy < 0.1 cm^3^	0.0 cm^3^	V2700cGy < 0.1 cm^3^	0.0 cm^3^
	Dmean < 3000 cGy	121.0 cGy	Dmean < 3000 cGy	118.0 cGy
BrachialPlex_L	V2400cGy < 0.1 cm^3^	0.0 cm^3^	V2400cGy < 0.1 cm^3^	0.0 cm^3^
Chestwall	V3000cGy < 30 cm^3^	0.0 cm^3^	V3000cGy < 30 cm^3^	4.939 cm^3^
Lungs	V2000cGy < 10%	0.00%	V2000cGy < 10%	0.00%
	V1000cGy < 15%	0.01%	V1000cGy < 15%	1.23%
	V500cGy < 20%	1.45%	V500cGy < 20%	3.30%
	Dmean < 500 cGy	76.8 cGy	Dmean < 500 cGy	139.5 cGy
Heart	V3500cGy < 0.1 cm^3^	0.0 cm^3^	V3500cGy < 0.1 cm^3^	0.0 cm^3^
	Dmax < 3500 cGy	3237.7 cGy	Dmax < 3500 cGy	3246.6 cGy
BrachialPlex_R	Dmax < 6600 cGy	12.5 cGy	—	—
Active myocardium	—	—	Dmean < 1000 cGy	910.6 cGy
Trachea	—	—	V3000cGy < 0.01 cm^3^	0.0 cm^3^

The second case involved a 61-year-old female diagnosed with thymic squamous cell carcinoma of the anterior mediastinum in May 2020. In August 2020, the patient underwent chemoradiotherapy, receiving a total dose of 66 Gy (2.2 Gy per fraction), followed by immunotherapy. A subsequent PET/CT scan in the follow-up identified an anterior pericardial mass, further confirmed by echocardiogram and contrasted cardiac MR images in November 2021. Contrasted CT indicated the pericardial mass increased from 26 mm x 18 mm in November to 36 mm x 23 mm in December. Given the high risks associated with surgery in the tumor location, the patient was recommended for SBRT treatment on the MR-Linac in January 2022. The SBRT treatment plan was designed accounting for the prior thymic radiation. Three months post-treatment, the patient’s chest pain was resolved. A CT chest with contrast suggested responsiveness of the pericardial mass and no progression of the original thymic carcinoma, as indicated in **Figure 3**. The centrally located anterior pericardial presumed metastasis remained controlled, along with the primary treatment-related changes in the anterior mediastinum, without evidence of local recurrence. A PET/CT scan acquired 6 months post-treatment revealed a reduction in size of the anterior pericardial metastasis, together with increased superior pericardial thickening. However, she was found to have recurrences outside of the SBRT field including a new 12 mm anterior pericardial and a 11 mm left lower lobe lung nodule. The new pericardial mass was separate from the treated cardiac metastasis. Both of new nodules were found to be within the radiation field of the primary mediastinum treatment. She also developed radiation pneumonitis and was treated with a long taper of high dose prednisone. Unfortunately, the patient had an acute myocardial infarction and died 8 months after her radiation treatment. This was felt to be unrelated to her SBRT treatment given her significant pre-existing significant coronary artery disease with history of coronary artery bypass surgery, stenting, and angioplasty.

## 4 Discussion

While cardiac metastases exhibit a higher incidence than primary cardiac tumors, they remain a rare disease. Currently, there is no standard for the treatment of cardiac metastases, and cardiac resection is infrequently recommended due to associated high risks and poor outcomes. Palliative interventions, chemotherapy, or radiotherapy (e.g., 24 Gy in 8 fractions) have been considered as treatment options (9, 10), yet these approaches demonstrate suboptimal prognosis. The emergence of SBRT and MR-guided adaptive radiotherapy (MRgRT) has positioned radiotherapy as a curative option, providing patients with precise and highly conformal radiation treatments. Conventional CT scans lack sufficient imaging capabilities for accurate identification of cardiac tumors and surrounding cardiac structures. In contrast, the MR-linac system offers superior soft-tissue contrast, reducing uncertainties in target delineation and enhancing sparing of adjacent tissues. In addition, the daily verification of patient anatomy and adaptability of treatment plans enable dose escalation with acceptable acute toxicities. Further gains in the image quality and cardiac motion management can be made by the application of breath holding or beam gating techniques. MR-guided SBRT has found application in several cases involving primary (11–13) or metastatic cardiac tumors (14), with dose regimens ranging from 30-40 Gy in 5 fractions to 60 Gy in 12 fractions, achieving promising treatment responses and no acute adverse events. The reported histology includes angiosarcoma, primary and recurrent sarcoma, uveal, desmoplastic, and cutaneous melanoma, as well as breast intraductal carcinoma. Notably, both undifferentiated carcinoma (19.5%) and squamous cell carcinoma (18.2%) have high rates of cardiac metastasis (2). To the best knowledge of the authors, this study presents the first instance of patients with neuroendocrine carcinoma and squamous cell carcinoma cardiac metastases being treated using SBRT on a 1.5 Telsa MR-Linac. Although follow up for our patients was limited, MR-guided SBRT was delivered successfully with tumor regression and minimal acute toxicity suggesting that this may be a feasible treatment approach for cardiac metastases.

## 5 Conclusions

We demonstrate the feasibility of using an MR-guided workflow for the treatment of cardiac metastases, yielding well-tolerated toxicities accompanied by symptomatic relief. Nevertheless, a more extensive dataset and extended follow-up periods are imperative for a comprehensive exploration of treatment efficacy. The growing interest and ongoing trials in the realm of hypo-fractionated SBRT delivered via MR-Linac promise avenue for addressing inoperable cardiac metastases. Continued research in this direction holds potential for refining treatment strategies and advancing patient outcomes.

## Data availability statement

The raw data supporting the conclusions of this article will be made available by the authors, without undue reservation.

## Ethics statement

The studies involving humans were approved by UT MD Anderson Cancer Center Institutional Review Board. The studies were conducted in accordance with the local legislation and institutional requirements. Written informed consent for participation was not required from the participants or the participants’ legal guardians/next of kin in accordance with the national legislation and institutional requirements. Written informed consent was not obtained from the individual(s) for the publication of any potentially identifiable images or data included in this article because This is a retrospective study covered by an institutional review board (IRB)-approved protocol (2022-0521). Informed patient consent is waived in this protocol due to the nature of a retrospective study.

## Author contributions

XC: Conceptualization, Data curation, Investigation, Methodology, Validation, Visualization, Writing – original draft. JW: Conceptualization, Data curation, Investigation, Methodology, Validation, Visualization, Writing – review & editing. AS: Data curation, Investigation, Validation, Writing – review & editing. BL: Data curation, Investigation, Writing – review & editing, Validation. NH: Data curation, Investigation, Validation, Writing – review & editing. MM: Data curation, Investigation, Validation, Writing – review & editing. YZ: Data curation, Investigation, Validation, Writing – review & editing. XW: Validation, Writing – review & editing. XZ: Validation, Writing – review & editing. JN: Validation, Writing – review & editing. SS: Validation, Writing – review & editing. LC: Supervision, Validation, Writing – review & editing. ZL: Supervision, Validation, Writing – review & editing, Funding acquisition. PL: Writing – review & editing, Funding acquisition, Resources, Supervision, Validation. JY: Writing – review & editing, Funding acquisition, Resources, Supervision, Validation.
